# Co-inhibition of epidermal growth factor receptor and insulin-like growth factor receptor 1 enhances radiosensitivity in human breast cancer cells

**DOI:** 10.1186/1471-2407-13-297

**Published:** 2013-06-19

**Authors:** Ping Li, Marlon R Veldwijk, Qing Zhang, Zhao-bin Li, Wen-cai Xu, Shen Fu

**Affiliations:** 1 Department of Radiation Oncology, Sixth People’s Hospital of Jiao Tong University, 600 Yi Shan Rd., Shanghai 200233, People’s Republic of China; 2 Department of Radiation Oncology, University Medical Center Mannheim, University of Heidelberg, Mannheim, Germany

**Keywords:** Epidermal Growth Factor Receptor (EGFR), Insulin-like Growth Factor Receptor 1 (IGF-1R), Radiosensitivity, Breast cancer, Co-inhibition

## Abstract

**Background:**

Over-expression of epidermal growth factor receptor (EGFR) or insulin-like growth factor-1 receptor (IGF-1R) have been shown to closely correlate with radioresistance of breast cancer cells. This study aimed to investigate the impact of co-inhibition of EGFR and IGF-1R on the radiosensitivity of two breast cancer cells with different profiles of EGFR and IGF-1R expression.

**Methods:**

The MCF-7 (EGFR +/−, IGF-1R +++) and MDA-MB-468 (EGFR +++, IGF-1R +++) breast cancer cell lines were used. Radiosensitizing effects were determined by colony formation assay. Apoptosis and cell cycle distribution were measured by flow cytometry. Phospho-Akt and phospho-Erk1/2 were quantified by western blot. In vivo studies were conducted using MDA-MB-468 cells xenografted in nu/nu mice.

**Results:**

In MDA-MB-468 cells, the inhibition of IGF-1R upregulated the p-EGFR expression. Either EGFR (AG1478) or IGF-1R inhibitor (AG1024) radiosensitized MDA-MB-468 cells. In MCF-7 cells, radiosensitivity was enhanced by AG1024, but not by AG1478. Synergistical radiosensitizing effect was observed by co-inhibition of EGFR and IGF-1R only in MDA-MB-468 cells with a DMF_10%_ of 1.90. The co-inhibition plus irradiation significantly induced more apoptosis and arrested the cells at G0/G1 phase in MDA-MB-468 cells. Only co-inhibition of EGFR and IGF-1R synergistically diminished the expression of p-Akt and p-Erk1/2 in MDA-MB-468 cells. In vivo studies further verified the radiosensitizing effects by co-inhibition of both pathways in a MDA-MB-468 xenograft model.

**Conclusion:**

Our data suggested that co-inhibition of EGFR and IGF-1R synergistically radiosensitized breast cancer cells with both EGFR and IGF-1R high expression. The approach may have an important therapeutic implication in the treatment of breast cancer patients with high expression of EGFR and IGF-1R.

## Background

Currently, breast cancer is the most common malignancy among women worldwide. Radiotherapy is considered mandatory for most patients undergoing breast-conserving surgery and appropriate for women at high risk of recurrence after mastectomy, but the locoregional control of breast cancer patients still is disappointed, especially in some subtypes like basal-like breast cancer [1]. The patients with basal-like breast cancer are associated with a high risk of local-regional failure compared to other subtypes [1,2]. One of the features of the patients is that they have abnormal signaling transduction pathways like epidermal growth factor receptor (EGFR) and insulin like growth factor 1 receptor (IGF-1R) [3,4]. These receptor tyrosine kinases have been implicated in radioresistance of breast cancer in preclinical and clinical studies [3,5,6], therefore, the combination of targeted therapy with radiotherapy has been investigated to improve local control rates [5].

Clinical studies of EGFR inhibitors might aid in the clinical introduction of anti-IGF-1R targeting strategies. Interactions between IGF-IR and EGFR signaling pathways have been previously described [7]. The interaction exists on multiple levels, either through a direct association between the two receptors, or indirectly via common interaction partners such as downstream effectors [7]. Sensitivity to EGFR inhibition has been linked to acquired mutations in the ATP binding site of the EGFR kinase domain and to increased IGF signaling, Co-inhibition of EGFR and IGF-1R has been found to cause synergy in growth inhibition and apoptosis induction in human breast cancer cells [8]. Considering the interplay between the IGF-1 and EGF systems and their role in the modulation of radiosensitivity, targeting multiple signaling pathways may maximize the response to irradiation.

Synergistic radiosensitization has been achieved by co-inhibition of multi-targets [9]. However, there are no reports about the impacts of co-inhibition of EGFR and IGR-1R on radiosensitivity of breast cancer cells. In this study, we aimed to investigate whether co-inhibition of EGFR and IGF-1R enhances the radiosensitivity of breast cancer cells with different expression of the two receptors, and also to assess the potential molecular mechanisms.

## Methods

### Cell lines and culture

The human breast cancer cell lines MDA-MB-468 (basal-like cell line with high expression of EGFR and IGF-1R) and MCF-7 (luminal-like cell line with high expression of IGF-1R, but low expression of EGFR) were used in this study [10], and purchased from the American Type Culture Collection (ATCC, Manassas, VA). The cells were cultured in Eagle’s MEM supplemented with 10% fetal bovine serum and 1% penicillin-streptomycin (GIBIC).

### Reagents

All antibodies were purchased from the Cell Signaling Technology, Inc (Danvers MA). The selective EGFR tyrosine kinase inhibitor (AG1478) and the IGF-1R tyrosine kinase inhibitor (AG1024) were purchased from Calbiochem (La Jolla CA). The inhibitors were dissolved in DMSO to prepare a 10 mM stock solution.

### Irradiation

Cells in a monolayer were irradiated at room temperature using 6MV X-rays from linear accelerators (Siemens, Germany) with dose rate of 3 Gy/min. A 1.5-cm bolus was used as a compensator

### Cell viability assay

Cells were incubated in the presence of serial increasing concentrations of AG1478 or AG1024 for 48 h. Then, 20 μM of MTT solution (5 mg/ml) was added into each well for 4 h. The reaction was stopped by removal of MTT, and 150 μl DMSO was added into each well, and then the plates were read at 570 nm. Percentage of cell viability was determined relative to control. Each experiment was done in six replicate wells for each drug concentration. All experiments were done in triplicate. The IC50 values were calculated with the SPSS software using bliss method.

### Colony formation assay

10^5^ Cells were seeded in 60 mm culture dishes, twenty- four hours later cells were treated with 10 μM AG1478 or/and 10 μM AG1024, control group received DMSO in the same concentration for 1 hour. Then cells were irradiated with single dose at 0 to 10 Gy with 6MV x rays. At 48 hours post-irradiation, the cells were detached from dishes with trypsin, and were seeded at various dilutions into 60 mm dishes in normal medium. The cells were cultured for 14 days. Each result was the average of at least three independent experiments. Colonies (>50 cells/colony) were fixed and stained with crystal violet. Survival curves were fitted by the linear-quadratic model using the Graphpad prism soft (version 5.0). Dose-modifying factor at 10% survival cells (DMF_10%_) were determined by taking the ratio of the radiation doses at the 10% survival level.

### Apoptosis and cell cycle assay by flow cytometry

Cells were treated with inhibitors (10 μM) for 1 h and were irradiated with 4Gy. They were harvested and washed with PBS at 48 hours after treatment. They were stained with propidium iodide (PI) and Annexin V (KeyGEN, Inc. Nanjing, China) for 10-20 min, and were detected by flow cytometry (Beckman Coulter, Inc.). For the analyses of cell cycle, the treated cells were fixed in 70% ethanol and stored at −20°C overnight; the cells were labeled with propidium iodide (50 μg/ml) and RNase (100 μg/ml) for 30 min before the analyses by flow cytometry with Multi-cycle system software package.

### Western blot analysis

MDA-MB-468 cells were exposed to 10 μM of AG1478 and/or 10 μM of AG1024 for 1 hour, and then incubated with the inhibitors after irradiated at 4Gy. After incubation for 24 hours, the cells were lysed and separated by sodium dodecyl sulfate polyacrylamide gel electrophoresis and transferred to polyvinylidene fluoride membrane, the membrane were incubated overnight with primary antibodies at 4°C with gentle shaking, and then were incubated for 2 h with horseradish peroxidase–labeled secondary antibody. All membranes were detected using the ECL plus chemifluorescent reagent (Amersham Biosciences). The extent of protein expression were quantified by the ImageJ soft from NIH [11] and normalized by the value of control expression in each group.

### In vivo studies

Female athymic nu/nu mice (4 to 6 weeks old) were obtained from laboratory animal center of Shanghai institutes for biological sciences, Chinese Academy of Sciences (Permit Number: SCXK 2007–005). All animal studies were strictly in accordance with a protocol approved by Ethic Committee for Animal Experimentation of Shanghai Jiaotong University. 5 × 10^6^ MDA-MB-468 cells were injected into the flanks of female athymic nu/nu mice. The mice with tumor volume 100 mm^3^ were randomly divided into five groups (5 mice/group), and treated with variable strategies. AG1478 were intraperitoneally injected with 10 mg/kg three times per week for 2 weeks and AG1024 were intraperitoneally injected with 1.5 mg/kg once per day for 2 weeks. Mice were irradiated 30 min after injection of inhibitors with 8 Gy on the first day. Tumor volume for xenografts was determined by a caliper and was calculated as volume = length × width^2^/2, where the width is the smallest measurement and the length is the longest measurement [12].

### Statistical analysis

Each experiments were performed in triplicate. For comparison of the difference between two groups, Student’s *t* test was used. For comparison of the difference between more than two groups, One-way ANOVA, Bonferroni were employed for statistical analysis using SPSS 11.0 for windows software. p values <0.05 were considered as statistically significant.

## Results

### The impact of inhibition of EGFR or IGF-1R on the cell viability

**Figure 1:**
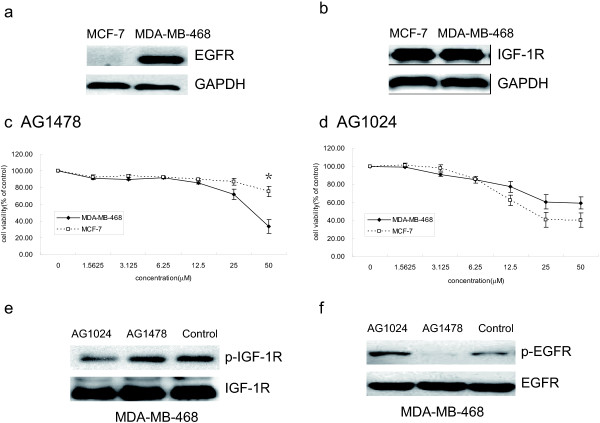
**Specific inhibition of EGFR by AG1478 or IGF-1R by AG1024. (a-b)** Under basal growth conditions, whole-cell extracts obtained from MDA-MB-468 and MCF-7 cells were analyzed for EGFR **(a)** and IGF-1R **(b)** expressions. **(c-d)** Cellular viability was measured by MTT assay. MDA-MB-468 and MCF-7 cells were treated with the indicated concentrations of AG1478 **(c)** (Students’ *t*-test, p = 0.022 at concentration of 50 μM) or AG1024 **(d)** for 48 h. **(e-f)** MDA-MB-468 cells were treated with 10 μM AG1478 or 10 μM AG1024 for 24 h. Western blot analysis was done on MDA-MB-468 cell lysates using antibodies specific for IGF-1R, p-IGF-1R **(e)** and EGFR, p-EGFR (f). * p < 0.05, MDA-MB-468 cells vs. MCF-7 cells.

MDA-MB-468 and MCF-7 cells have similar expression of IGF-1R, but EGFR was more expressed in MDA-MB-468 cells compared with MCF-7 cells (Figure 1a-b). Compared with MCF-7 cells, MDA-MB-468 were more sensitive to EGFR inhibitor AG1478 (IC50 to MDA-MB-468 and MCF-7 cells were 40.92 μM and 159.24 μM, respectively) as shown in Figure 1c. However, MCF-7 cells were found to be more sensitive to IGF-1R inhibitor AG1024 as compared to MDA-MB-468 cells (IC50 to MDA-MB-468 and MCF-7 cells were 58.75 μM and 24.91 μM, respectively) (Figure 1d), Interestingly, AG1024 that downregulated the expression of p-IGF-1R in MDA-MB-468 cells (Figure 1e), resulted into the upregulation of p-EGFR without influencing the levels of total EGFR (Figure 1f).

### Co-inhibition of EGFR and IGF-1R synergistically enhanced the radiosensitizing effect in MDA-MB-468 cells but not in MCF-7 cells

**Figure 2:**
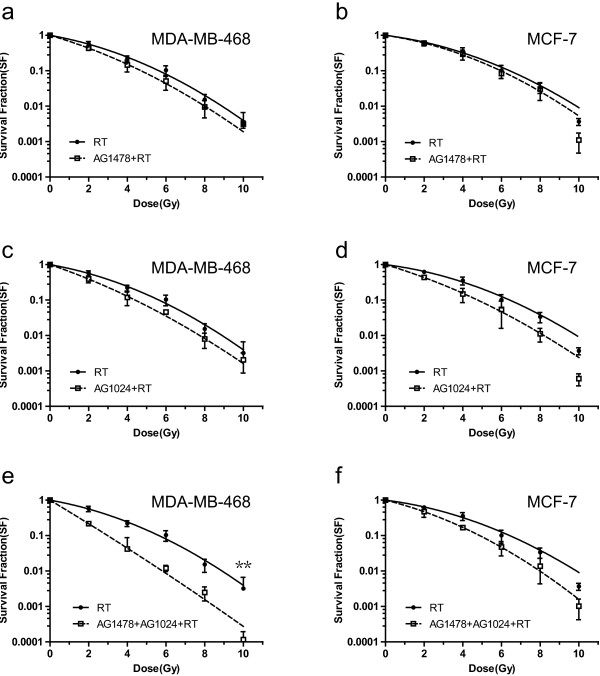
**Effect of AG1478 or/and AG1024 on radiosensitivity in MDA-MB-468 and MCF-7 cells.** MDA-MB-468 and MCF-7 cells were treated with the following inhibitors: DMSO in same concentration (as control), 10 μM AG1478 **(a, b)**, 10 μM AG1024 **(c, d)**, or their combination **(e, f)** for 1 h. After irradiation with indicated dose for 48 h, cells were trypsinized, counted, seeded at different dilutions and incubated for 14 days. Vertical bars represent standard deviation. Points, mean values from three independent experiments. (One-way ANOVA _MDA-MB-468_, F = 4.568, p = 0.038 at dose of 10Gy). ** p < 0.05. AG1478 + AG1024 + RT group vs. Other treated groups. RT = radiotherapy.

As shown in Figure 2, AG1478 moderately enhanced the radiosensitivity of MDA-MB-468 cells at all radiation doses, with a DMF_10%_ of 1.20, but not of MCF-7 cells (DMF_10%_ of 1.08). AG1024 sensitized both MDA-MB-468 and MCF-7 cells to radiation, with a DMF_10%_ of 1.28, 1.34, respectively. The radiosensitizing effect was further enhanced by the co-inhibition of EGFR and IGF-1R, with a DMF_10%_ of 1.90 in MDA-MB-468 cells, but not in MCF-7 cells (DMF_10%_ of 1.32).

### Co-inhibition of EGFR and IGF-1R combined with irradiation induced more apoptosis in MDA-MB-468 cells not in MCF-7 cells

**Figure 3:**
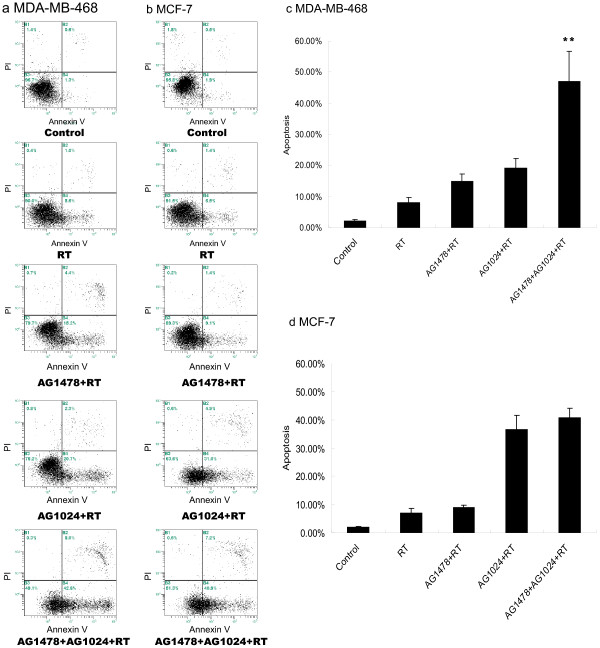
**Effect of AG1478 or/and AG1024 plus irradiation on apoptosis in MDA-MB-468 and MCF-7 cells.** Cells were exposed to DMSO in same concentration (as control), AG1478 10 μM, AG1024 10 μM or their combination combined with irradiation at dose of 4 Gy. Results are the mean value of three experiments, columns, mean; bars, S.D. (One-way ANOVA_MDA-MB-468_ F = 48.194, p < 0.001), ** *p* < 0.05, AG1478 + AG1024 + RT group vs. other treated groups. **(a-b)**: Representative picture of the apoptosis on MDA-MB-468 **(a)** and MCF-7 **(b)** cells. **(c-d)** Graphs show the percentage of apoptosis on MDA-MB-468 **(c)** and MCF-7 **(d)** cells. RT = radiotherapy.

As shown in Figure 3, either AG1478 or AG1024 combined with irradiation moderately induced apoptotic cells in MDA-MB-468 compared to irradiation alone (*p* = 0.016, *p* = 0.005, respectively). Concordant with MTT assays, no such induced apoptosis was observed by AG1478 plus irradiation in MCF-7 cells compared with irradiation alone (*p* = 0.141). However, AG1024 plus irradiation induced more apoptotic cells in MCF-7 cells (*p* = 0.001). While the cells were treated with both inhibitors plus irradiation, significant induction of apoptosis was seen in MDA-MB-468 cells. However, the combination of both inhibitors with irradiation in MCF-7 cells did not result in further increased apoptosis relative to treatment with AG1024 plus irradiation.

### Co-inhibition of EGFR and IGF-1R combined with irradiation significantly induced G0/G1 arrest in MDA-MB-468 cells

**Figure 4:**
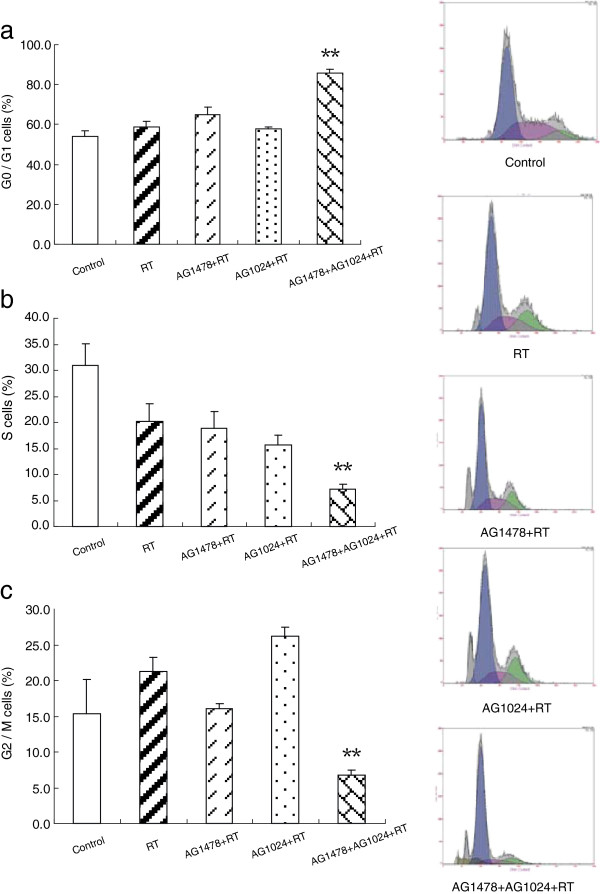
**Effect of AG1478 or/and AG1024 plus irradiation on cell cycle in MDA-MB-468 cells.** MDA-MB-468 cells were exposed to AG1478 10 μM, AG1024 10 μM and their combination plus irradiation at dose of 4 Gy for 48 h. Cell distribution in G0/G1 **(a)**, S **(b)**, G2/M **(c)** phase. Data represent mean values from three independent experiments. *Columns*, mean from three repeated experiments; *bars*, SD. (One-way ANOVA_G0/G1_ F = 71.498, p < 0.001; One-way ANOVA_S_, F = 6.897, p = 0.006; One-way ANOVA_G2/M_, F = 12.389, p = 0.001) ** p < 0.05, AG1478 + AG1024 + RT group vs. other treated groups. RT = radiotherapy.

As shown in Figure 4, a significant increase in G0/G1 phase cells after treatment with AG1478 combined with irradiation (*p* = 0.015, RT vs. RT plus AG1478) could be observed. But treatment of AG1024 combined with irradiation did not induced an accumulation of cells in the G0/G1 phase (*p* = 0.404, RT vs. RT plus AG1024). Unexpectedly, combined treatment with AG1478 and AG1024 plus irradiation resulted in a significant accumulation in the G0/G1 phase in more than 80% of the cells and a significant decrease of S and G2/M phase cells to less than 8% (*p* < 0.05, compared with other treated groups).

### Enhancement of the radiosensitizing effect of MDA-MB-468 cells through synergistical downregulation of Akt and Erk1/2

**Figure 5:**
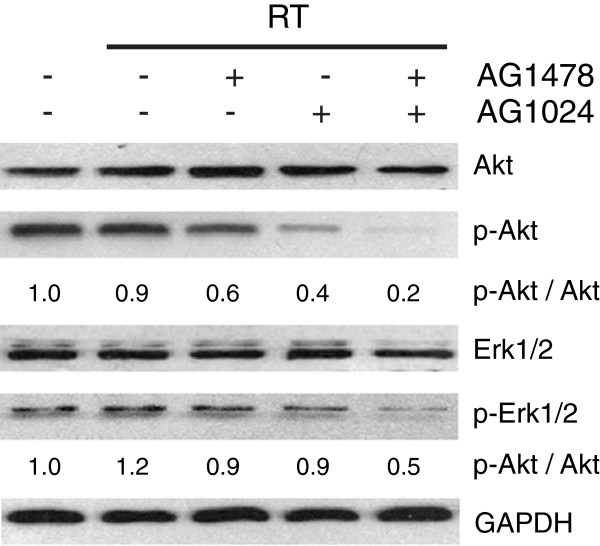
**Effect of AG1478 or/and AG1024 plus irradiation on total and phosphor-Akt and -Erk1/2 in MDA-MB-468 cells.** MDA-MB-468 cells were exposed to AG1478 (10 μM for 1 hour) and/or AG1024 (10 μM for 1 hour), and were irradiated at 4Gy for 24 hours. Total and phosphorylated Akt, total and phosphorylated Erk1/2 were examined. The density of each band were shown under the p-Akt and p- Erk1/2 panel, these represent phosphor-protein relative to total protein, the phosphor-protein/total protein values were then normalized to the value for control cells, the cells were assigned the value = 1. RT = radiotherapy.

As shown in Figure 5, when MDA-MB-468 cells were treated with AG1478 or AG1024 plus irradiation for 24 h, p-Akt level was partially reduced, but p-Akt was fully diminished by the combination plus irradiation. On the other hand, AG1478 or AG1024 plus irradiation had minimal influence on p-Erk1/2 expression in MDA-MB-468 cell lines, combining both inhibitors with irradiation could significantly decreased the expression of p-Erk.

### Co-inhibition of EGFR and IGF-1R combined with irradiation significantly inhibits MDA-MB-468 xenograft growth

**Figure 6:**
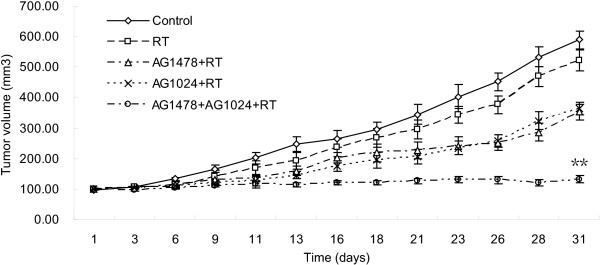
**In vivo radiosensitization of AG1478 or/and AG1024 in a nu/nu MDA-MB-468 xenograft model.** The treatment began when mean tumor volume reached 100 mm^3^, mice treated with DMSO, AG1478, AG1024, or a combination. AG1478 were intraperitoneally injected with 10 mg/kg three times per week for 2 weeks and AG1024 were intraperitoneally injected with 1.5 mg/kg once per day for 2 weeks. Irradiation with 8 Gy was given 30 min after drugs treatment on the first day. (One-way ANOVA F = 101.86, p < 0.001) ** p < 0.05, AG1478 + AG1024 + RT group vs. other treated groups. RT = radiotherapy.

As shown in Figure 6, the in vivo studies of co-inhibition of EGFR and IGF-1R on the anti-tumor effect of radiotherapy were determined in a nu/nu MDA-MB-468 xenograft mouse model. The irradiation group had minimal effects on tumor growth delay compared with control group. Either AG1478 or AG1024 combined with irradiation could inhibit the tumor growth compared with irradiation alone (*p* < 0.001). Compared with those two treatments, combining AG1478 and AG1024 with irradiation led to the most significant inhibition of tumor growth (p < 0.001) at day 31 post treatment.

## Discussion

EGFR and IGF-1R are commonly overexpressed in a significant number of cancers, included breast cancer [13,14], and its overexpression have been implicated to influence the response to irradiation in breast cancer cells [15]. There were about 65% with the overexpression of EGFR and 22.5% with the overexpression of IGF-1R in basal-like breast cancer patients [4,6]. The abnormal expression of those receptors have been observed to be associated with poor prognosis and unfavorable response to radiotherapy [6]. Since there were a cross-talk between EGFR and IGF-1R pathways and the cross-talk may be one of reasons for the resistance of cancer cells to drug and radiotherapy [16,17], co-inhibition of both pathways have been investigated and found out that it could synergistically inhibit tumor proliferation and growth [7,15]. Therefore, we hypothesized that co-inhibition of EGFR and IGF-1R would further impact the response of breast cancer cells to irradiation.

In our studies, the different response to irradiation after co-inhibition of EGFR and IGF-1R in MDA-MB-468 and MCF-7 cells adds to the evidence that both signaling pathways may be involved in the treatment response. Firstly, the radiosensitizing effect by either EGFR or IGF-1R inhibitor depended on the expression level of EGFR and IGF-1R in both cells. Secondly, inhibition of IGF-1R resulted in a slight upregulation of p-EGFR in MDA-MB-468 cells, which corroborates the study by other reports [15,18]. Furthermore, both cell lines had a different sensitivity to AG1024 although both cell lines had similar expression level of IGF-1R (Figure 1b). Those findings supported that there were the interaction between EGFR and IGF-1R. Co-inhibition of EGFR and IGF-1R plus irradiation resulted in significantly increased apoptosis and mitotic death relative to any single inhibitor plus irradiation in MDA-MB-468 cells. In addition, in vivo studies further verify the radiosensitizing effects by co-inhibition of EGFR and IGF-1R in MDA-MB-468 xenografts. These results added the evidence that both EGFR and IGF-1R may be involved in the regulation of radiosensitivity, the response to radiotherapy in breast cancer like basal-like subtype may be improved by co-targeting EGFR and IGF-IR.

The possible mechanism for synergistical radiosensitizing effect by co-targeting EGFR and IGF-IR may be associated with their collective downstream pathways -- PI3K/Akt and Ras/Raf/MAPK, both pathways involved in the regulation of radiosensitivity through the downstream proteins Akt and Erk1/2 [19,20]. It has been reported that inhibition of PI3K/Akt signaling pathway led to radiosensitize the tumor cell by affecting repair of DNA double-strand breaks via DNA-PKcs, and this pathway inactivates Bad and caspase-9 and activates p21, p27 and Mre11, which are associated with cellular radiosensitivity [21,22]. Activated Erk1/2 has also been observed to confer radioresistance in breast cancer cells [19]. Inhibition of both Akt and Erk1/2 may achieve synergistic radiosensitization in some subtypes of cancer cells. In present study, we found that co-inhibition of EGFR and IGF-1R could completely abolished the p-Akt and p-Erk1/2 and resulted in a synergistic radiosensitizing effect in MDA-MB-468 cells. These results suggested that co-targeting EGFR and IGF-1R radiosensitized the MDA-MB-468 cells through both PI3K/Akt and MAPK signaling pathways.

In addition to the potential of growth factor inhibitors to reverse pro-survival signal, they may also sensitize cells to irradiation by altering cell cycle control. The growth factor inhibitors have been shown to induce G0/G1 arrest, and this alteration redistributes cells from relatively radioresistant S phase to more sensitive phase like late G1 or G2/M [23]. On the other hand, although tumor cells arrest at some checkpoints in order to repair radiation-induced damage, it require growth factors to proceed effectively [24], therefore, inhibition of growth factor receptor make the process unable to facilitate repair, contributing to cell death. Our data show that co-targeting EGFR and IGF-1R plus irradiation significantly reduced S phase and arrest cells at G0/G1 phase in MDA-MB-468 cells, profound tumor cell kill was observed, therefore, the cells were sensitized to irradiation.

## Conclusion

In summary, both in vitro and in vivo studies support that synergistic radiosensitizing effect by co-inhibition of both pathways mainly through the synergistic downregulation of p-Akt and p-Erk1/2. Our results suggest that the strategy of block more than one pathway holds promise to enhance the radiosensitivity of some subtypes breast cancer, but it is critical to evaluate the profile of expression of EGFR and IGF-1R in breast cancer patients before the strategy is applied into the clinical setting.

## Competing interests

The authors declare that they have no competing interests.

## Authors’ contributions

Li P, Veldwijk MR and Xu WC performed the cell culture, western blot and animal experiments, Li P and Zhang Q carried out the data analysis, Li ZB participated in irradiation study. Li P, Veldwijk MR and Shen Fu drafted the manuscript, Shen Fu designed the study, and the research funds were supported by Shen Fu. All authors read and approved the final manuscript.

## Pre-publication history

The pre-publication history for this paper can be accessed here:

http://www.biomedcentral.com/1471-2407/13/297/prepub
